# The American Public Health Association

**Published:** 1886-01

**Authors:** 


THE AMERICAN PUBLIC HEALTH ASSOCIATION has become one of the permanent institutions of the country. Its inception dates back only a dozen years, but in that short time it has collected and published in its annual volumes a large amount of practical and most valuable information upon all subjects connected with public health. It is not a medical society, nor is it a society of sanitary engineers, nor is it made up of health-officers. In fact it is an association where any one, who takes an interest in the problems affecting the physical well-being of society, may meet and attempt, at least, to solve these problems. No pathy, no creed, no code, no sex, no nationality excludes any one from membership or office. It attempts to do for the American people what health-congresses and similar national organizations are trying to do for the peoples of the old world. Such measures and such studies on the part of the non-professional public are peculiar to this last half of this nineteenth century. In former generations it was fashionable to despise the body and look only after the welfare of the soul. It is true, there was an old maxim that "cleanliness is next to godliness", but it was accepted as a figurative statement, or as articles in the Confession of Faith —doctrinal, not practical. It is a sign of better things, for the body at least, that from all parts of the United States and from Canada, such a large number of persons could think it worth while to visit Washington at the last annual meeting of the Association, and spend four days in the discussion of these great health-questions. We note with pleasure that the next meeting will be held in Toronto, Canada.

